# The Medical Visit to Paris

**Published:** 1905-05-20

**Authors:** 


### Hospital Clinics.

### THE MEDICAL VISIT TO PARIS.

The visit of English medical men to Paris has been a great success. It was undertaken as a return for the visit made by the French physicians and surgeons to London in October, and if on that occasion our French guests were entertained a tithe as well or learnt a fraction as much as the English have done in Paris the labours of the executive are amply repaid. The hospitality and friendship of our hosts knew no bounds. The main party of Englishmen left London at nine o'clock on Wednesday morning, were welcomed by a deputation at Calais and were received officially at the Gare du Nord in Paris. In the evening there was a reception at the Sorbonne by M. Liard, vice rector of the University of Paris. The proceedings were somewhat curtailed by the failure of the electric light which led to their being conducted by the help of a few candles which just served to make the darkness visible. The return of light, however, enabled the visitors to view the magnificent amphitheatre and to discover the sumptuous repast which the committee had provided. Speeches were made or read on behalf of the English visitors by Sir William Broadbent, Professor Clifford Allbutt and Dr. George Ogilvie.

On Thursday a cloudless sky, a pleasant breeze and uninterrupted sunshine, combined to make Paris look its very best. Two-and-a-half inches of rain in 60 hours last week had cleared the atmosphere, laid the dust, and brought the horse-chestnuts and laburnums to perfection. The day was passed in an agreeable mixture of labour and entertainment. The hospitals were all in full work, and showed both their excellences and defects. The hospitalities, public as well as private, were lavish, and by many delicate attentions the French showed the qualities which have always rendered them such charming friends. The most impressive gathering perhaps, because it was the least imposing, was the reception given by Monsieur Mesureur, the director of the general administration of the Assistance Publique. Here in a room which was plain almost to austerity, and which contrasted all the more sharply with the magnificent salons, the brilliant reception and the excellent band at the Hôtel de Ville, was all that made the French system of hospitals differ from the English plan. In France the hospital system is centralised; in England it is a system of isolated units. Here were the men who quietly and unostentatiously directed the Assistance Publique of Paris, dealing with the medical requirements of a very large population. From them we have much to learn in the matter of organisation; from us they may receive details of considerable importance in the matter of housing, ventilation, and construction. They greet us cordially, realising that there is no progress in medical science which is not at once a benefit to the sick of both nations.

The morning of the second day was devoted to the examination of the hospitals, and everywhere the guests were kindly and even enthusiastically received by their hosts, who replied in the fullest manner to innumerable questions. Friday afternoon was devoted to an excursion to Chantilly, the magnificent château left by the Duc d'Aumale to the French nation as represented by the Académie de France. It is situated about the same distance from Paris as Windsor is from London, and is full of the choicest treasures of art. Two special trains took the party to Chantilly, and carriages conveyed them through the beautiful forest green with the verdure of early spring, past the great racecourse and the stables which make Chantilly a name of repute throughout the world, and many a heart was touched by the pack of foxhounds marshalled to do honour to the Englishmen. Saturday proved itself as full a day both in work and pleasure as were Thursday and Friday. In the afternoon a solemn and touching ceremony took place—a visit of everyone to the Institut Pasteur, and a formal tribute from the profession of medicine in England to the greatness, now happily universally acknowledged throughout the world, of the genius of Pasteur. Madame Pasteur was present after the reception, and it was delightful to see and to admire her to whose care, affection, and support the entire world owes so much. Her strength carried Pasteur through the dark days of trial and disappointment, when as yet his labours were unrecognised; her constant affection and continued work gave him a happy home which enabled him to give his whole time to the great thoughts which occupied his brain. In the fulness of time she now has her reward, and the great but reverent crowd which filed through the chapel, and, by the hand of Dr. Kingston Fowler laid flowers on the grave of Pasteur, gave testimony to the honour in which he is held, and to the deathlessness of his renown amongst the fellow countrymen of Lister, who shares with him the position of a saviour of the human race. Of this visit the French, with their usual courtesy, gave each visitor a lasting token in the form of a copy of Roty's bronze medal, with an excellent bust of Pasteur in profile on the obverse.

The great banquet was held in the evening at the Grand Hotel. It was honoured by the presence of the English Ambassador. More than 450 sat down, and although the speeches were inaudible for the most part, the "*bon*" or "*fire*" of the French and the cheers of the English made up in noise what was lacking in words. The investiture of Sir William Broadbent with the Cross of the Legion of Honour brought the proceedings to a fitting termination, and testified once again, but we hope not for the last time, to the warmth of feeling which exists between the members of the profession in France and England. Both nations are greatly indebted to Dr. Sillonville who first initiated the enterprise of a professional reunion, to Dr. Triboulet, Dr. Lucas-Championnière, Sir William Broadbent, Dr. Dawson Williams, Dr. Jobson Horne, and the members of the French and English committees who have carried it to so successful an issue. Lastly, Madame Triboulet and the ladies who worked with her have placed the English ladies under a deep debt of gratitude and have shown them how great is the organising power and the capability of French women, for if the stay of the Englishmen was made pleasant in Paris, the visit of the English women will ever live in their memories as a holiday, all too short indeed, but filled with the little surprises and delicate attentions to which our nation is indeed quite unaccustomed.

Now, what has been gained in actual knowledge by our visit to Paris? We have learnt, in the first place, that the stores, drugs, and food required by the various hospitals can be cheaply and efficiently distributed by a central organisation, which can vary the amount and quality according to the temporary requirements of each institution. There is no reason to prevent such an arrangement being entered into mutually by the various hospitals in London and in the large towns in the provinces. Secondly, we are taught that we are quite unduly extravagant in the matter of dressings and equipment, both in our wards and operating theatres. The Paris hospitals have gone somewhat to the other extreme, not for want of taste or knowledge, but literally for want of means. The poor are treated as poor, and are not nursed in unaccustomed surroundings, so that the bareness of the walls and the scantiness of the furniture does not appear to them so obvious as it would do to ourselves. The beginnings of an improved system of nursing is already apparent, and, when we remember that it is less than 50 years since our hospitals were dependent upon a class of women similar to that now employed in the majority of French hospitals, we may reasonably suppose that trained nurses of our own type will soon become general in France. With a few exceptions, the cubic space allotted to the beds and the ward ventilation is markedly defective, but this is remedied in some measure by the ample space allotted to the hospitals themselves. Paris is a city of vast open spaces and green trees, just as Rome is a city of magnificent fountains, and the cramped buildings of the hospitals in London compare most unfavourably with the fine forecourt and spacious quadrangles of the older charitable institutions of Paris. From the purely professional side there is also much to learn. The surgeons are obliged to admit into their wards all the cases allotted to them, and in the general hospitals like the Hôtel-Dieu it happens therefore that hysterectomies and other cases usually allotted to specialists in England come constantly under their care. They draw, too, from much larger groups of population, and the aggregate of cases passing through their wards is larger than it is with us.

It is very profitable that the working members of the medical profession in each country should exchange visits, and we trust that the series thus begun may be long continued. A cordial friendship has sprung up between the members who have attended the meetings, and we may hope that this cordiality will be still further improved by the working of the newly founded society which is to be unique in having no subscriptions and is known as L'entente cordiale Médicale. Frenchmen having once come to London will now know that there are no insuperable difficulties in the way, for the *entente cordiale* will make the way easy, and it is to be hoped that the French ladies who vied with each other to make the visit of the English ladies a perfect success will no longer fear to cross the Channel, and on the next occasion, choosing an earlier season in the year, will find that London has attractions and is sometimes free from the fogs they dread so much.

